# Retinal Changes in Early-Onset cblC Methylmalonic Acidemia Identified Through Expanded Newborn Screening: Highlights from a Case Study and Literature Review

**DOI:** 10.3390/genes16060635

**Published:** 2025-05-25

**Authors:** Paola Michieletto, Francesco Baldo, Maurizio Madonia, Luisa Zupin, Stefano Pensiero, Maria Teresa Bonati

**Affiliations:** Institute for Maternal and Child Health “Burlo Garofolo”, 34137 Trieste, TS, Italy

**Keywords:** tandem mass spectrometry biomarkers, epi-cblC, infantile maculopathy, BEM, sensory nystagmus, motor nystagmus

## Abstract

Background: Methylmalonic acidemia combined with homocystinuria (cblC) can lead to infantile maculopathy. Although significant visual deterioration is commonly reported in early-onset cblC, we found poor awareness regarding formal assessments of ocular complications, especially in newborns, and of how these complications relate to the timing of therapy initiation. In this work, we present our experience and perform a literature review. Methods: We performed sequential fundus examinations, optical coherence tomography (OCT) and full-field electroretinography (ERG) under sedation following detection of signs of retinal degeneration. We also assessed visual fields using kinetic attraction perimetry. Results: We report a newborn who was referred on the eighth day of life, following a diagnosis of cblC through newborn screening (NBS), and who began treatment that same day. Close monitoring of retinal changes through fundus examinations allowed the detection of signs of retinal degeneration at 3 months, which progressed when checked at 5 months. At 7 months, OCT showed retinal thinning with the appearance of bull’s eye maculopathy in the corresponding region on fundoscopy; ERG revealed a reduction in the amplitude of both scotopic and photopic components, whereas kinetic attraction perimetry showed no abnormalities. Genetic investigation confirmed the disease, compound heterozygous for a nonsense variant in *MMACHC* and a splicing one in *PRDX1*. Conclusions: In cblC, retinal degeneration occurs in the first months of life despite timely treatment and adequate biochemical control, and it may manifest before any signs of visual deprivation appear. However, there is an early, narrow window during which therapy may slow down retinal degeneration enough to prevent sensory nystagmus. We recommend initiating therapy immediately after biochemical diagnosis, along with close ophthalmological monitoring, before the appearance of any signs.

## 1. Introduction

Combined methylmalonic acidemia and homocystinuria (OMIM #277400, MAHCC), also known as cblC, is the most common inborn error of intracellular cobalamin (vitamin B_12_) metabolism, with a prevalence of 1:37,000 to 1:100,000. In Italy, cblC was introduced in newborn screening (NBS) between 2016 and 2017 [1]. NBS allows detection of patients, often before the onset of symptoms, and early treatment.

CblC results from a deficiency of the MMACHC chaperone, which impairs the conversion of vitamin B_12_ into its two active forms, adenosylcobalamin and methylcobalamin. These serve as cofactors for two enzymes, methylmalonyl-CoA mutase (which converts methylmalonyl-CoA into succinyl-CoA in mitochondria) and methionine synthase (which catalyzes in the cytosol the conversion of homocysteine into methionine), whose dysfunction leads to elevated levels of methylmalonic acid (MMA) and homocysteine (Hcy) in plasma and urine, along with normal or reduced plasma methionine (Met) levels [2].

The disorder is inherited in an autosomal recessive manner. It can be either monogenic, with bi-allelic pathogenic variants in *MMACHC*, or digenic, with one of the two pathogenic variants located in the adjacent *PRDX1* gene, encoding peroxiredoxin 1, involved in cell defense against oxidative stress with effect on cell growth, differentiation and apoptosis. Phenotypically, digenic cblC is nearly indistinguishable from the monogenic form, as pathogenic variants in *PRDX1*—of splicing type—lead to aberrant *PRDX1* antisense activity, causing cys-hypermethylation of the *MMACHC* promoter and subsequent transcriptional silencing [3]. Since this mutational event results from an epigenetic modification at the *MMACHC* locus, digenic cblC is called ‘epi-cblC’.

CblC can present with a broad spectrum of clinical features and age of presentation. Patients with early-onset disease (<12 months) may have feeding difficulties, failure to thrive, developmental delay, hypotonia, seizures, anemia, thrombocytopenia, neutropenia, and microangiopathy. Late-onset disease presents after the first year of life with neurologic dysfunction such as cognitive decline, psychiatric disturbances, and subacute degeneration of the spinal cord. In the recently recognized prenatal form, the symptoms may appear during pregnancy or shortly after birth and include fetal growth retardation, and neurological and developmental issues.

Patients with prenatal and early-onset cblC may have significant ocular features, whereas visual loss and ocular complications are rare and mild in the late-onset form. The typical pattern of retinal degeneration is characterized by bilateral macular atrophy with a bull’s-eye pattern, i.e., a hypopigmented perimacular zone surrounded by a hyperpigmented ring. These marks can be seen on fundoscopy and optical coherence tomography (OCT). In addition to maculopathy, the ophthalmic phenotype includes pigmentary retinopathy and, less frequently, optic atrophy. Ocular findings are strabismus, nystagmus and/or visual inattention [2,4].

Therapy cannot entirely prevent ocular symptoms, even in patients identified by NBS or prenatal diagnosis and biochemical abnormalities aggressively treated early/in utero. Although the natural history of cblC is well known—with visual deterioration and blindness being common outcomes in early-onset patients—there is still limited awareness regarding the formal assessment of ocular complications, particularly timing and the types of ophthalmological evaluations required [4]. Typically, an ophthalmological examination is only recommended at diagnosis [2].

We report on the onset and progression of ocular disease measured by ERG and OCT in a seemingly healthy little girl who underwent treatment after biochemical diagnosis of a cobalamin defect at NBS. Prompted by the patient’s history and a review of cases with early-onset cblC and ocular involvement, we realized that certain ophthalmological features, such as nystagmus, should be further categorized to assess the impact of early therapeutic intervention. We recommend that neonates with a positive NBS for MMA and confirmed hyperhomocysteinemia begin treatment without delay and be promptly referred to a pediatric ophthalmologist experienced in inherited metabolic disorders.

## 2. Materials and Methods

### 2.1. Patient

Based on NBS results, the newborn patient was suspected of having a cobalamin C/D defect and subsequently referred to the Neonatology Department to the Pediatric Clinic of Maternal and Child Health IRCCS Burlo Garofolo for further investigation and treatment. Visual and ophthalmologic evaluations were carried out at the hospital’s Ophthalmology Department.

### 2.2. Newborn Screening and Confirmatory Testing

NBS samples of dried blood spots (DBSs) were collected between 48 and 72 h, according to the recommended time frame. Blood levels of propionylcarnitine (C3), MMA and Hcy were measured by tandem mass spectrometry at the University Hospital of Padova. Second DBSs were requested within 24 h upon abnormal first-screening results [1]; in this second test, the second-tier marker included Met. Plasma Hcy, acylcarnitines, MMA and Met were measured to confirm the diagnosis further. Plasma vitamin B_12_ levels were determined to rule out a maternal deficiency.

### 2.3. Molecular Analysis

Whole exome sequencing (WES) of the proband and parents (trio WES) was performed at the Medical Genetics Laboratory of IRCCS Burlo Garofolo as described in Feresin et al. [5].

### 2.4. Visual and Ophthalmologic Testing

We performed optical coherence tomography (OCT) and full-field electroretinography (ERG) under sedation, and measured the extension of the binocular visual field by means of ‘Attraction perimetry’, an advanced evolution of the Metrovision (MonCv3) system, designed to detect and quantify early campimetric alterations in glaucoma [6]. The binocular attraction visual field was performed using Goldmann V4 stimuli and a background brightness of 10 candles/m^2^.

### 2.5. Literature Review

We reviewed pathogenic variants with the support of online databases, including ClinVar (https://www.ncbi.nlm.nih.gov/clinvar/, accessed on 18 March 2025), and HGMD^®^ Professional (https://digitalinsights.qiagen.com/) (accessed on 18 March 2025).

Moreover, we extracted data from case reports and case series of patients affected by infantile cblC to compare retinal disease features, treatment initiation and outcome with those of our patient. PubMed search terms included ‘maculopathy’, ‘ocular’, ‘retinal’, and ‘retinopathy’. Each was combined with the search term ‘cblc type methylmalonic acidemia’ (accessed on 6 February 2025). We excluded from the review investigations focusing on late-onset cblC, as well as animal and in vitro studies.

## 3. Results

### 3.1. Clinical Report

The patient was the first offspring of healthy unrelated Italian parents of Caucasian ethnicity and was born at term by vaginal delivery after normal pregnancy. The mother’s first pregnancy resulted in termination due to gastroschisis; the second pregnancy resulted in spontaneous termination in the second trimester. Family history was otherwise not contributory. Her birth weight was 2790 g (10–25th centile), birth length 49 cm (25–50th centile), and occipitofrontal circumference (OFC) 33 cm (10–25th centile).

On the eighth day after birth, she was referred for study following biochemical diagnosis of cblC/cblD defect by NBS (Table 1). The patient exhibited slight drowsiness and poor sucking, resulting in mild dehydration as suggested by dry skin (at the turgor test, it took the skin 3 s to return to its original position) and mucous membranes, and decreased diuresis collected in the anamnesis. At the neurological examination she was found to have moderate axial and peripheral hypotonia. She was started on intramuscular hydroxocobalamin (OHCbl) 1 mg a day (1 mg/mL solution), after drawing for blood tests (Table 1) and having ruled out maternal vitamin B_12_ deficiency. Biochemical tests also included blood count, capillary blood gas analysis and urine stick, which were found average, and ammonium dosage, which was 113 µg/dL (nv 27–90). The baby promptly recovered, and starting the following day, betaine 100 mg was administered three times daily, along with folic acid 5 mg twice a week. Otoemissions, automated auditory brainstem responses (AABR), electrocardiogram, echocardiogram, and transfontanellar ultrasound performed at 9 and 13 days of age, were all normal.

She underwent fundus examination on the ninth day which was normal. At the age of 3 months, she did not exhibit any pupillary defects nor strabismus or alterations in ocular motility. She had a good fixation and normal pursuit. Cycloplegic refraction in dioptres was slightly astigmatic (+2.00 cyl/70° in the RE and +1.50 cyl/100 TABO in the LE). Fundus examination revealed a mild pigmentary perifoveal dystrophy with a standard optic disc.

**Figure 1:**
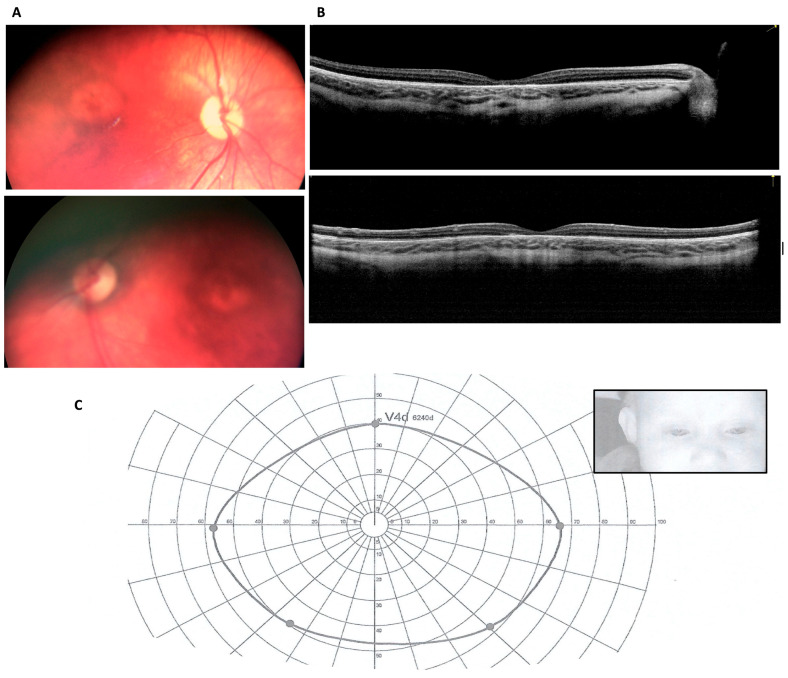
Bilateral bull’s eye maculopathy (BEM) as detected at fundus examination and OCT scans performed in the corresponding region at 7 months. (**A**). Bilateral BEM is characterized by a round hypopigmented central zone surrounded by a hyperpigmented ring. Top image: right eye (RE); bottom image: left eye (LE). (**B**). OCT images of the macula. A very thin fovea (total foveal thickness of 72 µm in RE and 78 µm in LE) is visible in both eyes due to the development of foveal atrophy involving only the outer retinal layers. Top image: RE, oblique scan; bottom image: LE, vertical scan. (**C**). The binocular attraction visual field shows a normal extension for the age. The infrared image of the child’s face during the examination is shown in the top right window and allows us to identify the fixation changes made by both eyes and the head.

At 5 months, the fundus examination showed central macular atrophy with only slightly pale optic disc. At 7 months the evaluation was performed under sedation: a bilateral bull’s eye maculopathy was present (Figure 1A); OCT images (Optovue iVue, Freemont, CA, USA) (Figure 1B) showed a very thin fovea with an Outer Plexiform Layer (OPL) that became even thinner when moving toward the fovea, until it disappeared in the atrophic area that involved only the external layers. The full-field ERG showed an amplitude reduction of both scotopic and photopic components. We performed a binocular Kinetic attraction visual field (Figure 1C) some days later, resulting in a standard (bilateral temporal extension of 60°).

At the last follow-up, at the age of 10 months, her psychomotor development was within normal limits. As for growth parameters, weight was 7.4 kg (3rd centile), length 70.5 cm (27th centile), and OFC 43 cm (9th centile).

### 3.2. Biochemical Features

As shown in Table 1, with therapy Hcy values gradually decreased to slightly above the normal range; C3 and MMA values averaged and Met increased. These data document the achievement of a good biochemical control. OHCbl was administered by a subcutaneous catheter device [7] for 4 months, with success, to avoid multiple punctures. We removed the subcutaneous injection port due to the parents’ concern about infections at the injection site, and intramuscular therapy was resumed.

### 3.3. Genetic Findings

The patient was found to be compound heterozygous for the pathogenic variants *MMACHC* (NM_015506.3): c.331C>T, p.(Arg111*) [8,9] in exon 3 and *PRDX1* (NM_002574.4): c.515-1G>T [3,9], inherited from the father and the mother, respectively. Genetic findings fit with the patient’s biochemical and clinical features.

The *MMACHC* (NM_015506.3): c.331C>T is a nonsense substitution that truncates the protein at codon 111, which is 172 amino acids from the end of the protein. The nonsense variant was classified as pathogenic according to the ACGS/ACMG-AMP criteria PVS1, PM2_moderate, PP3 and PP5_supporting (https://wintervar.wglab.org/, accessed on 30 December 2024).

The *PRDX1* (NM_002574.4): c.515-1G>T is a splicing variant that activates a cryptic acceptor site in intron 5, producing the skipping of the last exon (6) and the loss of transcription termination signal of *PRDX1* [3].

### 3.4. Digenic Methylmalonic Acidemia and Homocystinuria, cblC Type (epi-cblC)

In epi-cblC, the subjects are compound heterozygotes for a pathogenic variant in an allele of the disease-causing gene (*MMACHC*) and a secondary epimutation in the promoter of the other allele of *MMACHC*. The epimutation results from a splicing defect in the *PRDX1* gene, caused either by c.515-1G>T, as seen in our patient, or by c.515-2A>T, which are located in the canonical splice acceptor site in intron 5.

**Figure 2:**
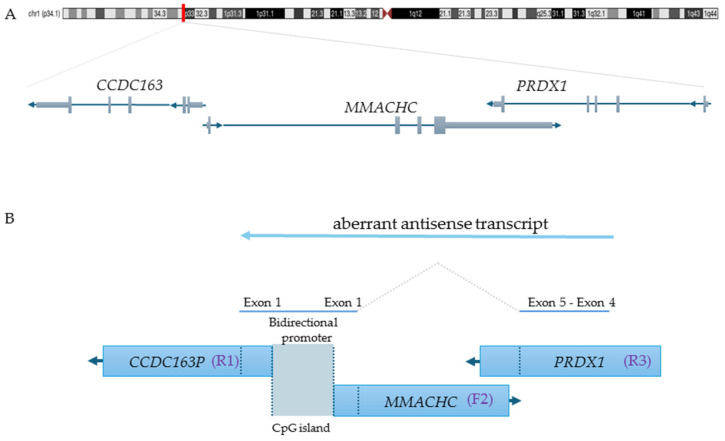
Genomic architecture of the epi-cblC gene trio and aberrant antisense transcription leading to *MMACHC* promoter epimutation. (**A**). The chromosome location of *MMACHC*, *CCDC163P* and *PRDX1* is shown along with their transcription orientations (UCSC Genome Browser Genome viewing, hg38). Specifically, *CCDC163P* is reverse 1 (R1), *MMACHC* is forward 2 (F2), and *PRDX1* is also reverse 3 (R3). (**B**) The picture depicts the mechanism of formation of the prolonged antisense transcript derived from the *PRDX1* splicing variant. Additionally, it highlights the location of the CpG island that is subject to methylation [3].

*MMACHC* belongs to a gene trio on the 1p34.1 chromosome region, in which it is a sense gene flanked by *CCDC163P* (a pseudogene) and *PRDX1* in the opposite orientation (trio reverse(R1)/forward(F2)/reverse(R3)) (Figure 2A).

Each *PRDX1* splicing variant extends the antisense transcription across *PRDX1* exons 4 and 5, part of *MMACHC* exon 1, the bidirectional promoter of the adjacent *MMACHC/CCDC163P* genes, and part of the first exon of *CCDC163P,* as a result of the activation of a cryptic antisense splicing site within the *MMACHC* exon 1 (Figure 2B). This aberrant transcription may cause RNA polymerase collision and the formation of triplexes, leading to the methylation of the gene promoter and the consequent silencing of the gene expression [3,9].

Although epi-cblC is characterized by compound epigenetic-genetic heterozygosity with haploinsufficiency of *MMACHC*, it is classified as digenic. This arises from the inability to exclude the possibility that *PRDX1* splicing variants may have deleterious effect on peroxiredoxin 1, thereby potentially affecting the disease course.

As a unique example among human diseases, in cblC, the *MMACHC* epimutation is inherited in cis with the pathogenic splicing variant of *PRDX1* [3].

### 3.5. Literature Mining

The [*MMACHC* (NM_015506.3): c.331C>T, p.(Arg111*); *PRDX1* (NM_002574.4): c.515-1G>T] genotype has been described in a male from Northern Italy –10 years at last follow-up– with early-onset disease, who started treatment with enteral feeding, red blood cells transfusions, OHCbl, L-carnitine, betaine and folates from 2 months, when he was diagnosed after a clinical/biochemical assessment [9]. The boy was born before the implementation of NBS, so a precocious diagnosis, prior to symptom onset, was not possible. Briefly, his clinical history began on the fourth day with sucking difficulties and recurrent vomiting, then severe weight loss on the twenty-first day. He underwent hospitalization because of severe failure to thrive and vomiting with subsequent drowsiness, hypotonia, sepsis, respiratory distress and acute neurological crisis between 42 and 104 days after birth. A brain MRI at age 2 months showed frontotemporal atrophy with hygromas. He exhibited nystagmus, macular hypoplasia, and exotropia/exophoria at 4 months, 6 and 7 years, respectively. At the age of 3 years, he was diagnosed with a moderate intellectual disability [9].

In cblC a genotype-phenotype correlation has not been identified. However, the genotype homozygous for the common c.271dupA allele is characterized by early-onset phenotype and early childhood-onset maculopathy. Moreover, the presence of homozygous or compound heterozygous frameshift and non-sense genetic variants are often associated with a more severe phenotype.

Through the PubMed search, we retrieved 15 manuscripts [4,8,10,11,12,13,14,15,16,17,18,19,20,21]. Some of them were excluded from the discussion because they dealt with late-onset cblC [12,18] and animal [19] or in vitro [17] disease models. In particular, for the study, the manuscripts by Weisfeld-Adams et al. [4] and Ku et al. [11] were especially valuable, since they reported when patients were started on therapy and included those treated prenatally.

Indeed, prenatal molecular diagnosis—through target sequencing of pathogenic variants in both copies of *MMACHC* or one copy of both *MMACHC* and *PRDX1* using DNA extracted from chorionic villi or amniocytes—may be proposed where an older sibling is affected. Additionally, intramuscular hydroxocobalamin may be administered to the mother during the third trimester of pregnancy if pathogenic variants are identified in prenatal diagnosis.

Alternatively, couples may be offered preimplantation genetic diagnosis through assisted reproductive technology.

Table 2 displays the patients selected for comparison and discussion: we included early-onset cblC patients who began therapy within the first month of life, also considering in this category those reported as having initiated treatment ‘immediately after diagnosis’.

## 4. Discussion

In newborns or infants, ocular involvement, especially retinal degeneration, is frequent among the earliest detectable signs of early-onset cblC type methylmalonic acidemia with homocystinuria. As in the infant here described, macular atrophy may be the first sign observed, even without evidence of visual deprivation, such as nystagmus. Therefore, in cblC macular atrophy may be overlooked if not actively sought, for instance through OCT, since it can detect even the most subtle and asymptomatic changes of maculopathy.

Furthermore, by exhibiting macular atrophy as early as 5 months of age, despite prompt initiation of therapy and adequate biochemical control, this clinical report provides further evidence of the very early onset of retinal disease in epi-cblC-type methylmalonic acidemia [9].

Conversely, macular atrophy may be overlooked when systemic issues, such as hypotonia and poor feeding, may take precedence over a detailed eye examination.

Some authors have hypothesized that maculopathy in infantile cblC likely develops before birth.

The exact mechanism of the specific eye involvement in cblC remains unclear. Since a critical phase of foveal development occurs postnatally, extending through infancy into early childhood, the fovea may be particularly vulnerable to the toxic accumulation of homocysteine and methylmalonic acid during this window, potentially explaining the rapid progression of macular alteration observed in affected patients [4,14]. Additionally, ocular tissues are particularly vulnerable to metabolic disturbance due to their high energy requirement. A deficiency in the MMACHC protein impairs the intracellular processing of vitamin B_12_ into adenosylcobalamin, a cofactor for the methylmalonyl-CoA mutase. This mitochondrial enzyme is a component of the propionate catabolic pathway, which metabolizes branched-chain amino acids, odd-chain fatty acids and the side chain of cholesterol. Methylmalonyl-CoA mutase catalyzes the conversion of methylmalonyl-CoA into succinyl-CoA that enters the tricarboxylic acid cycle, serving as an anapleurotic substrate [26].

In the outer retina, photoreceptors, retinal pigment epithelium and Müller cells possess a high density of mitochondria to support their metabolic activity. Furthermore, retinal ganglion cells have unmyelinated axons until they pass through the lamina cibrosa. In this unmyelinated region, the generation and propagation of action potentials demand substantial energy supplied by a high mitochondrial content [27]. Moreover, photo-oxidative stress makes ocular tissues more susceptible to oxidative damage [28]. Furthermore, although according to the Human Protein Atlas *MMACHC* is not specifically expressed in the retina compared to other tissues [29], the Human Eye Transcriptome Atlas shows an enrichment of *MMACHC* expression in the choroid and retinal pigment epithelium, suggesting, for this gene, a potential role in retinal function [30].

Eye involvement may be indirect. A post-mortem histopathological study of a cblC infant revealed, at the electron microscopy level, diffuse storage of material in cytoplasmic vacuoles across various ocular tissues, along with swollen mitochondria in the corneal epithelium and degenerated mitochondria in the iris pigment epithelium [31].

Macular changes can develop as early as before the first month of age (Table 2 and [17]), often presenting as subtle macular pigmentary or atrophic changes. These macular changes may progress to severe macular lesions at a dramatically fast space, usually within the first two years of age, but as early as 6 months of age (Table 2 and [17]).

Notably, the clinical findings in patient n. 56 [4] (Table 2) closely resemble those observed in our patient.

Among the patients listed in Table 2, five out of nineteen, including the patient reported here, exhibited retinal degeneration without developing nystagmus (Table 2). Furthermore, seven out of nineteen patients had retinal degeneration and nystagmus (Table 2), while another seven exhibited nystagmus without retinal degeneration (Table 2).

In early-onset retinal dystrophies, nystagmus is the most common sign, occurring, for instance, in 76% of the cohort of 50 children studied by Suppiej et al. [32], and it typically manifests within the first 6 months of life, as occurred in 34 children of the 40 who exhibited nystagmus from the same cohort [32]. The onset timing of nystagmus in retinal diseases is influenced by the severity and extent of tissue damage that occurs during the development of the fixation reflex. This reflex, which enables the eyes to reflexively orient and maintain fixation on a target, typically develops within the first three months of life. In the child here reported, the development of maculopathy after the third month of life allowed the patient to establish the fixation reflex, thereby preventing the onset of nystagmus and thus prospecting a more favourable visual outcome.

Sensory nystagmus, often of the pendular type in the early stages, can result from macular dystrophy, either congenital or occurring within the first 2–3 months of life, or congenital developmental abnormalities such as foveal or optic nerve hypoplasia.

The presence or absence of nystagmus in patients with early-onset maculopathy is related to the timing of maculopathy development relative to the fixation reflex. When maculopathy develops after the fixation reflex has already emerged, as observed in our patient, sensory nystagmus is unlikely to occur. This is consistent with sensory nystagmus patterns.

On the other hand, the development of nystagmus in the absence of maculopathy suggests the presence of different types of nystagmus in cblC patients. This aspect has not been addressed before. In fact, in the patient reported by Gerth et al. [33], nystagmus was present from birth and diagnosed as congenital motor nystagmus. Motor nystagmus, typically of the jerking type, unlike sensory nystagmus, can also be present at birth and may result from neurological abnormalities detectable at brain MRI. Additionally, congenital motor nystagmus may be idiopathic (without brain MRI abnormalities) and sometimes exhibit familiarity, usually an autosomal dominant inheritance pattern.

Therefore, it is crucial to distinguish early-onset cblC patients with nystagmus due to sensory alterations from those with motor nystagmus.

Early diagnosis and treatment, as in our patient, could influence the development of sensory nystagmus but would have no effect on motor nystagmus.

Interestingly, patient n. 6 described by Ku et al. [11] (Table 2), who was followed ophthalmologically from 8 months, showed signs of nystagmus at 8 months but developed the characteristic BEM maculopathy at 4.8 years of age. This clinical history suggests that a thorough ophthalmological follow-up is warranted for cblC patients, even after the first two years of life.

Similar to patient n. 6 [11], the fundus oculi of patient P3, as described by Schimel et al. [24] (Table 2), appeared normal upon examination at 6 months. However, evidence of photoreceptor disease was detected through ERG. Therefore, we suggest performing ERG in cblC patients even if they do not exhibit signs of maculopathy.

Retinal changes in cblC patients who underwent early treatment overlap with those diagnosed later. For example, the epi-cblC patient with the same genotype as our patient exhibited an ocular phenotype that falls between those of the first two patients listed in Table 2.

Optic atrophy may also be present in each of the three subgroups, often associated with a worse outcome. In cblC, optic atrophy may coexist with retinal disease or may present as an isolated finding [34]. In instances where optic atrophy is associated with retinal pathology, it is plausible that the atrophy results from diminished input from the retina. Specifically, in the presence of maculopathy, the optic nerve ganglion cells may undergo degeneration due to the absence of activation from their corresponding photoreceptors, particularly the cones. In our patient, it appears that the observed optic pallor is likely a secondary consequence of the underlying maculopathy. This assumption fits with the ERG findings. Conversely, it is also possible that the optic atrophy represents a primary condition, independent of retinal involvement.

Hydroxocobalamin supplementation is considered the standard therapy for cblC, with studies suggesting higher doses, especially early in treatment, may improve the biochemical and neurodevelopmental profiles. Indeed, vitamin B_12_ can cross the retinal blood barrier [35] and brain blood barrier [36], thus supporting the potential efficacy of hydroxocobalamin therapy.

However, an Italian study reported no improvement in maculopathy, with a median hydroxocobalamin dose of 0.55 mg/kg/day (range 0.30–0.90) compared to medium (0.09 mg/kg/day) and low (0.06 mg/kg/day) doses. In contrast, improvements in metabolic profile and neurocognitive outcome were found in the high dose group [37]. A Luxemburg study using higher doses (mean ± SD 6.5 ± 3.3 mg/kg/day) found better ophthalmologic and cognitive outcome in 4 infants who started treatment before 5 months of age, compared to a child who began treatment at 5 years [38]. Additionally, Venditti et al. [39] reported that five of the six patients treated with hydroxocobalamin dose intensification (ranging from 0.4 to 2.7 mg/kg/day) did not develop maculopathy or retinopathy. In contrast, within a historical cohort of 27 patients who received lower doses (≤0.3 mg/kg/day), all developed maculopathy.

The optimal dosing regimen for hydroxocobalamin in cblC has not yet been established. However, recent studies suggest that a higher dose may be more efficacious in biochemical control than a lower dose.

We suggest that neonates following a positive NBS for MMA with confirmed hyperhomocysteinemia be immediately started on cobalamin supplementation and promptly referred to a pediatric ophthalmologist with expertise in inherited metabolic diseases for early evaluation. Indeed, based on the above observations, we confirm that prompt treatment may benefit systemic disease control, although it does not prevent macular disease progression [16,20,40].

## 5. Conclusions

In conclusion, early-onset cobalamin C deficiency can present with significant ocular complications, such as retinal degeneration, nystagmus and optic neuropathy.

Ocular involvement may be ‘cryptic’ and can precede any systemic manifestations, making cblC a particularly insidious disease. Conversely, its onset may be delayed by years compared to the systemic onset, even if the latter occurs very early. Therefore, careful monitoring for ocular signs and symptoms is necessary throughout the course of development.

Pre-symptomatic diagnosis, primarily through newborn screening and genetic testing, allows for prompt initiation of treatment, typically involving vitamin B_12_ supplementation and other metabolic therapies, to manage the condition and mitigate its effects.

It is worth noting that genetic testing for methylmalonic acidurias must consider epi-cblC, and pathogenic variants must be searched for in *MMACHC* and *PRDX1*. Epivariation in R1/F2/R3 trios of genes is a disease pathomechanism to consider in patients exhibiting typical manifestations of a recessive disorder but bearing a single heterozygous pathogenic variant in the causal gene.

We recommend beginning therapy as soon as the biochemical defect is confirmed and prenatally in the case of a prenatal diagnosis.

In cblC there are two types of nystagmus: sensory, resulting from retinal degeneration and/or optic atrophy, and motor, which may arise from brain abnormalities or be idiopathic. Additionally, *MMACHC* itself may be a susceptibility gene for motor or congenital idiopathic nystagmus.

## Figures and Tables

**Table 1:** Biochemical features of the patient, from birth to her last follow-up, and treatment.

Time	C3 (µm/L)	MMA (µm/L)	Hcy (µm/L)	Met (µm/L)	Therapy
2 d (1st DBS)	10.05 (nv < 3.3)	66 (nv < 3.0)	51.15 (nv < 10)	-	/
5 d (2nd DBS)	08.41 (nv < 3.3)	101.6 (nv <3.0)	57 (nv < 10)	4 (nv 9–40)	/
8 d	7.76 (nv < 2.5)	75.6 (nv < 5)	230 (nv 5–15)	9 (nv 9–39)	/
9 d	-	-	240 (nv 5–15)	-	OHCbl im 1 mg, betaine 100 mg × 3 daily, folic acid 5 mg × 2 weekly
12 d	0.83 (nv < 2.5)	14.5 (nv < 5)	72 (nv 5–15)	27 (nv 9–39)	same as above
20 d	0.81 (nv < 2.5)	4.7 (nv < 5)	36 (nv 5–15)	25 (nv 9–39)	same as above
1 m 5 d	1.19 (nv < 2.5)	3.6 (nv < 5)	36 (nv 5–15)	47 (nv 9–39)	OHCbl (0.7 mL solution daily) with i-port advance^TM^, betaine 300 mg × 3 daily, folic acid 5 mg × 2 weekly
1 m 28 d	0.81 (nv < 2.5)	2.3 (nv < 5)	-	30 (nv 9–39)	same as above
3 m 1 d	-	-	28 (nv 5–15)	-	same as above
3 m 20 d	-	-	18 (nv 5–15)	-	same as above
5 m 9 d	0.46 (nv < 2.5)	1.5 (vn < 5)	15.7 (nv < 12)	19 (nv 9–39)	same as above
7m 11 d	-	-	21 (nv 5–15)	-	OHCbl im 5 mg (1 mL solution daily), betaine 400 mg × 3 daily, folic acid 5 mg × 2 weekly
10 m 1 d	-	-	24 (nv 5–15)	33 (nv 9–42)	OHCbl im 5.5 mg (1.5 mL solution daily), betaine 500 mg × 3 daily, folic acid 5 mg × 2 weekly

DBS, dried blood spot; C3, Propionylcarnitine; MMA, Methylmalonic acid; Met, Methionine; Hcy, homocysteine; OHCbl, hydroxocobalamin; m, month; d, day; nv, normal value.

**Table 2:** Comparison of patients with early-onset cblC presenting with ophthalmologic manifestations and receiving prompt initiation of therapy.

**a. Subgroup of Patients with Maculopathy Without Nystagmus**										
Age at First Retinal Exam	Retinal Findings (Fundus) and Age	OCT	ERG	Nyst	Strab	Optic Atrophy	Treatment Start Day	Visual Outcomes At Last Follow-Up	Study Design	Reference
9 d	Mild pigmentary perifoveal dystrophy (3 m); central macular atrophy, slightly pale optic disc (5 m); BEM (7 m)	Foveal thinning at OPL (7 m)	Reduction of scotopic and photopic components (7 m)	-	-	-	8	FF	Prospective	This study, F
4 m	BEM with progressive macular atrophy progressing between 4–7 m; peripheral non-perfusion	Progressive OPL and ONL atrophy to virtual disappearance of these layers in the fovea between 4–7 m	Abnormal	-	-	-	4	FF (8 m)	Retrospective and prospective	[4] (P56, M)
6 w	BEM at 6 w	NR	NR	-	-	-	8	FF (6 w)	Retrospective and prospective	[4] (P57, M)
6 m	Maculopathy	NR	Grossly normal at 12 m; diminished photopic and absent scotopic responses at 3 y; complete flattening at 10 y	-	NR	+	IAD	3/200 (10 y)	Retrospective	[8] (P8, M; P53 in [4])
6 m	Maculopathy (BEM at 6 m; bone spicules; progression of the macular lesions, new mid- and far-peripheral RPE mottling in a geographic fashion at 2.8 y; TDP and bone spicules at 9.8 y)	Staphyloma in the central area of the chorioretinal atrophy (9.8 y)	Significant decrease in scotopic responses, moderate decrease in photopic responses (9.8 y)	-	-	+ (9.8 y)	<1 m	20/150 (7.3 y)	Retrospective and prospective	[11] (P4, F)
**b. Subgroup of patients with maculopathy and nystagmus**										
Age at first retinal exam	Retinal findings (fundus) and age	OCT	ERG	Nyst	Strab	Optic atrophy	Treatment start day	Visual outcomes at last follow-up	Study design	Reference
1 m	Maculopathy	NR	Abnormal photopic and scotopic	+	NR	-	IAD	20/80 (10 y)	Retrospective	[8] (P2, M; P47 in [4])
7 m	Maculopathy (Faint BEM at 7 m, stable to 2 y)	NR	NR	+	-	-	Prenatal	CSM	Retrospective and prospective	[11] (P3, M)
NR	Maculopathy at 3 y	NR	NR	+	-	-	Prenatal	‘low vision’ (14 m)	Retrospective and prospective	[4] (P59, M)
NR	Maculopathy at 3 y	NR	NR	+	-	-	<1 m	20/100–20/50	Retrospective	[22] (M; P32 in [4])
NR	Maculopathy; RPE lucency and pigmentary retinopathy at 4 y	NR	NR	+	-	+	<1 m	CSM	Retrospective	[23] (P4, M; P36 in [4])
NR	Maculopathy; bilateral central retinal thinning with gray pigmentation at 4 y	NR	NR	+	+	-	<1 m	FF	Retrospective	[23] (P5, M; P37 in [4])
NR	Maculopathy with atrophy at 3 y; myopia	NR	NR	+	-	NR (poor VEP responses to flash and pattern stimulation)	<1 m	LA	Retrospective	[23] (P8, M; P40 in [4])
**c. Subgroup of patients with nystagmus without maculopathy**										
Age at first retinal exam	Retinal findings (fundus) and age	OCT	ERG	Nyst	Strab	Optic atrophy	Treatment start day	Visual outcomes at last follow-up	Study design	Reference
5 m	Unremarkable fundus	NR	NR	+	-	-	<1 m	CSM	Retrospective and prospective	[11] (P5, M)
8 m	BEM from 4.8 y, stable at last follow-up (7.3 y)	Parafoveal atrophy of outer retinal structures, consistent with BEM (4.8 y); worsening foveal and parafoveal atrophy of the outer retina and RPE, along with subretinal debris	Mildly decreased amplitude for rod and cone function (4.8 y, and stable at 6.8 y)	+ (from 8 m)	-	+ (5.4 y)	<1 m	20/125 (7.3 y)	Retrospective and prospective	[11] (P6)
6 m	Normal at 6 m	NR	Decreased photopically, extinguished scotopically	+ (intermittent, horizontal and pendular, with occasional vertical component)	-	-	<1 m	CSM (6 m)	Prospective	[24] (P3, F; P24 in [4])
24 m	Normal at 24 m. Absent maculopathy; peripheral ‘salt and pepper’ retinopathy, normal discs at 14 y	NR	Scotopic normal, photopic slightly abnormal (decreased b-wave amplitude) at 14 y	+ (Kestenbaum surgery at 5 y)	-	-	?	20/40 (14 y)	Retrospective	[25] (P3, M; P31 in [4])
NR	Absent maculopathy; mottling of RPE with small dark central clump of pigmentation (7 y)	NR	Normal	+	-	+	<1 m	FF (7 y)	Retrospective	[23] (P1, M; P33 [4])
NR	Normal at 3 y	NR	Normal	+	-	-	<1 m	FF (3 y)	Retrospective	[23] (P9, M; P41, in [4])
5 m	Normal at 11 y	NR	Normal	+	NR	+	IAD	20/100 (11 y)	Retrospective	[8] (P5, F; P50 in [4])

+, present; -, absent; NR, not reported/not done; P, patient; M, male; F, female; d, day; m, months; y, years; BEM, bull’s eye maculopathy; OPL, outer plexiform layer; ONL, outer nuclear layer; VEP, visual evoked potentials; FF, fix and follow; CSM, central, steady, maintained; LA, light adaptation; IAD, immediately after diagnosis; TDP, temporal disc pallor.

## Data Availability

Data are contained within the article.

## Abbreviations

The following abbreviations are used in this manuscript:

cblC	Methylmalonic acidemia combined with homocystinuria
BEM	Bull’s Eye Maculopathy
NBS	Newborn Screening
DBS	Dried Blood Spot
OCT	Optical Coherence Tomography
ERG	Electroretinography
C3	Propionylcarnitine
MMA	Methylmalonic acid
Hcy	Homocysteine
Met	Methionine
OHCbl	Hydroxocobalamin
OPL	Outer Plexiform Layer
ONL	Outer Nuclear Layer
FF	Fix and Follow
CSM	Central, Steady, Maintained
